# Oral Immune Priming Treatment Alters Microbiome Composition in the Red Flour Beetle *Tribolium castaneum*

**DOI:** 10.3389/fmicb.2022.793143

**Published:** 2022-04-13

**Authors:** Ana Korša, Lai Ka Lo, Shrey Gandhi, Corinna Bang, Joachim Kurtz

**Affiliations:** ^1^Institute for Evolution and Biodiversity, University of Münster, Münster, Germany; ^2^Department of Genetic Epidemiology, Institute of Human Genetics, University of Münster, Münster, Germany; ^3^Institute of Immunology, University of Münster, Münster, Germany; ^4^Institute of Clinical Molecular Biology, Christian-Albrecht University of Kiel, Kiel, Germany

**Keywords:** microbiome, oral immune priming, injection priming, bacteria, insect immunity, infection, *Tribolium castaneum*, *Bacillus*

## Abstract

It is now well-established that the microbiome is relevant for many of an organism’s properties and that its composition reacts dynamically to various conditions. The microbiome interacts with host immunity and can play important roles in the defenses against pathogens. In invertebrates, immune priming, that is, improved survival upon secondary exposure to a previously encountered pathogen, can be dependent upon the presence of the gut microbiome. However, it is currently unknown whether the microbiome changes upon priming treatment. We here addressed this question in a well-established model for immune priming, the red flour beetle *Tribolium castaneum* exposed to the entomopathogenic bacterium *Bacillus thuringiensis* (*Bt*). After priming treatments, the microbiota composition of beetle larvae was assessed by deep sequencing of the V1-V2 region of the bacterial 16S rRNA gene. We compared the effect of two established routes of priming treatments in this system: injection priming with heat-killed *Bt* and oral priming *via* ingestion of filtered sterilized bacterial spore culture supernatants. For oral priming, we used several strains of *Bt* known to vary in their ability to induce priming. Our study revealed changes in microbiome composition following the oral priming treatment with two different strains of *Bt*, only one of which (*Bt tenebrionis, Btt*) is known to lead to improved survival. In contrast, injection priming treatment with the same bacterial strain did not result in microbiome changes. Combined with the previous results indicating that oral priming with *Btt* depends on the larval microbiome, this suggests that certain members of the microbiome could be involved in forming an oral priming response in the red flour beetle.

## Introduction

Microbes and their hosts share a long and complex ecological and evolutionary history. They both form a dynamic interplay where host genetics can alter microbiome communities (Taylor and Vega, 2020), while members of the microbiome can protect the host against pathogens by releasing bacteriocins and toxins (McLaren and Callahan, 2020). Microbiomes have also been shown to be important in the development and activation of the host immune system (Hernández-Martínez et al., 2010; Koch and Schmid-Hempel, 2012; Onchuru et al., 2018). The hosts’ adaptive immunity can alter gut microbiota composition and diversity in mice (Zhang et al., 2015), while symbionts and microbes have been shown to play an important role in shaping innate immunity in honeybees (Horak et al., 2020) and to influence the infection success of a parasite in bumblebees (Mockler et al., 2018). Yet, their role in an important defense strategy of invertebrate hosts is far from understood: “immune priming” is a form of innate immune memory that leads to enhanced protection upon secondary infection (Kurtz and Franz, 2003; Little and Kraaijeveld, 2004). Over the last decade, this phenomenon has been described in numerous invertebrate species (for review, refer to Contreras-Garduno et al., 2016; Milutinović et al., 2016; Sheehan et al., 2020). Immune priming can occur within and across generations and shows similarities to trained immunity in vertebrates (Netea et al., 2011); however, the detailed mechanisms behind it remain largely unknown for most species (refer to Tetreau et al., 2019; Mondotte et al., 2020).

With an increasing number of studies trying to explore the nature of the priming response in insects, it turned out that the microbiome plays a role in priming in some model systems, but mechanisms are still unclear. For example, it has been shown that eradication of gut microbiota in adult *Anopheles gambiae* eliminated the immune priming against *Plasmodium falciparum* parasites (Rodrigues et al., 2010). However, in another study with *Anopheles albimanus* infected with *Plasmodium berghei*, priming did not depend on the presence of the microbiome (Contreras-Garduño et al., 2015). In our model organism, the red flour beetle *Tribolium castaneum*, the microbiome is necessary for forming a priming response that protects against oral infection with the bacterial pathogen *Bacillus thuringiensis tenebrionis* (Futo et al., 2016). These findings indicate that members of the microbial community of the host seem to contribute to the interplay between the host immune system and the pathogens. Nevertheless, a general explanation of how this might work is still missing.

The red flour beetle shows a specific priming response toward strains of the spore-forming entomopathogen *Bacillus thuringiensis* (*Bt*). This bacterium expresses plasmid-encoded crystalline inclusions (Cry toxins) specifically toxic to various insect orders after ingestion (Palma et al., 2014). In the red flour beetle, there are two different routes of infection with *Bt* that lead to different responses in gene expression and immune activity (Behrens et al., 2014). For septic wounding or injection, vegetative *Bt* cells are introduced into the body cavity (Roth et al., 2009; Ferro et al., 2019); for oral infection, *Bt* spores are ingested with the food (Milutinović et al., 2013). The oral route, which is often considered to be more natural, has been used less often for priming and infection experiments and seems to work only with beetle larvae (Milutinović et al., 2013). Like the infections, the priming response can be triggered *via* both septic and oral routes. Septic or injection priming is strain-specific, and treatment includes the use of heat-killed vegetative *Bt* cells that are introduced into the hemocoel *via* septic wounding or injection. This priming route has been demonstrated in the beetle larvae and adults and can be transferred to the offspring (Roth et al., 2009; Tate et al., 2017; Ferro et al., 2019; Schulz et al., 2019). Oral priming treatment works *via* ingestion of the sterile supernatant of the spore culture (Milutinović et al., 2014). Even though it is not clear what substance from the supernatant is responsible for the response, this form of priming also shows some degrees of strain specificity and, as mentioned above, requires microbiota (Futo et al., 2016, 2017). The oral priming treatment leads to the upregulation of immune recognition genes and elevated levels of reactive oxygen-based defenses, suggesting alertness of the immune system (Greenwood et al., 2017). Taken together, these findings indicate that there might be some sort of interaction of the hosts’ immune response with the microbiome, but it is not clear whether the formation of the oral priming response affects the resident microbial community. Given the described differences between the routes of infection and priming procedures in terms of immune responses and protection, it is important to know if they also influence the resident microbiome differently.

Here, we made use of 16S rRNA sequencing, to examine how the different routes of priming treatment, as well as the different bacterial strains used for oral priming, affect the microbiome composition of red flour beetle larvae. For microbiome analyses, we used isolated RNA instead of DNA. This method avoids amplification of chloroplast DNA (from flour) and enables assessing the microbial species that are metabolically active upon treatment. We studied the microbiome composition at two different time points, 24 h after and 4 days after the priming treatment based on the previously established priming and infection protocols (Roth et al., 2009; Milutinović et al., 2014; Ferro et al., 2019). We used the *Bacillus thuringiensis tenebrionis* (*Btt*) strain for both priming treatment routes, and additionally, for the oral priming route, two further *Bt* strains: *Bacillus thuringiensis tolworthi* (*Bt tolworthi*) that carries a different Cry toxin than *Btt*, and *Bt407*^–^, a strain that is deprived of the Cry toxin and does not lead to priming or mortality upon oral exposure (Milutinović et al., 2014, 2015). With this approach, we wanted to observe whether different priming treatments have any influence on the microbiome composition of the beetle larvae and thereby advance our understanding of the evolution of forms of immune memory within invertebrate immune defenses.

## Materials and Methods

### Model Organisms

In this study, we used wild-type *Tribolium castaneum* (Cro1) population which was collected from Croatia in 2010 (Milutinović et al., 2013) and adapted to laboratory conditions for numerous overlapping generations until the start of this experiment in 2019. Beetles were reared on organic wheat flour (Bio Weizenmehl Type 550, DM-drogerie markt GmbH + Co. KG) supplemented with 5% brewer’s yeast (flour mixture was previously heat-sterilized at 75°C for 24 h), at 30°C, 70% relative humidity, and 12-h/12-h light–dark cycle.

In all priming treatments, we used three different entomopathogenic gram-positive *B. thuringiensis* strains: *B. thuringiensis morrisoni* var. *tenebrionis* [*Btt*; BGSCID 4AA1 acquired from the Bacillus genetic stock center (BGSC)], *Bacillus thuringiensis tolworthi* [*Bt tolworthi*, Bacillus Genetic Stock Center (BGSC, Ohio State University, United States)], and *B. thuringiensis 407*- *(Bt407*-; kindly provided by Dr. Christina Nielsen-Leroux, Institut National de Recherche Agronomique, 78285 Guyancourt Cedex, France).

All of the bacterial strains were stored at 25% glycerol at –80°C before the experiment started.

### Experimental Design

**FIGURE 1:**
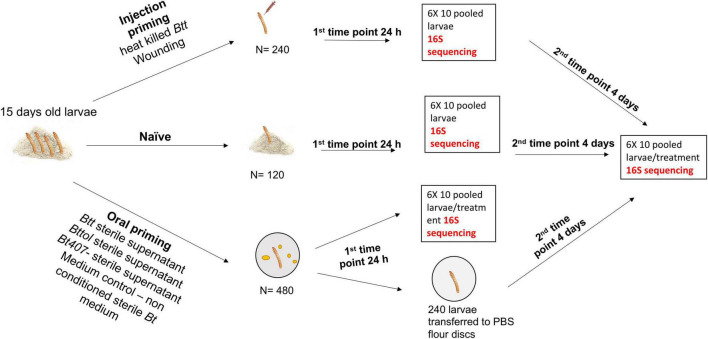
Overview of the experimental design and sample preparation for sequencing.

To assess the microbiome composition upon different routes of priming treatment, we performed injection and oral priming of larvae (Figure 1). Approximately 2,000 1-month-old adults were allowed to lay eggs for 24 h. Age-synchronized 15-day-old larvae were obtained from the beetle culture and used for all the priming experiments. Since we were interested in possible changes in the overall microbiome upon different treatments and not solely the gut microbiome composition, we processed whole larvae without dissection nor surface sterilization. The previous data showed that surface sterilization did not have a significant effect on the microbial load (Silva, 2017).

For injection priming, we had two treatments: “Injection_*Btt”* (i.e., injection of heat-killed *Btt*) and “Injection_Wounded” (i.e., injection of PBS). Moreover, a “Naïve” group served as a control (i.e., non-injected). Oral treatments consisted of dietary exposure of larvae to the filter-sterilized supernatant of one of the following bacterial strains: *Btt*, which carries the Cry3Aa toxin: “Oral_*Btt*”; *Bt tolworthi*, which carries the Cry3Ba toxin: “Oral_*Bttol*”; *Bt407^–^*, which does not produce any Cry toxin and does not lead to immune priming or mortality after ingestion: “Oral_*Bt407*^–^” (Milutinović et al., 2013, 2014). Moreover, exposure to sterile, non-conditioned medium served as a control: “Oral_Medium.” To examine whether there is a change in microbiome overtime after priming treatment, we sampled larvae at two different time points. The first time point corresponded to 24 h after priming treatment, where six replicates of ten pooled larvae per treatment were frozen in liquid nitrogen. The second time point corresponded to the time where larvae would have been challenged with bacteria, which is four days after priming when six replicates of ten pooled larvae per treatment were frozen in liquid nitrogen and kept at –80°C until RNA extraction. At both time points, we used six replicates of ten pooled naïve larvae (not treated, kept on flour). RNA was extracted in five batches, each batch consisting of all treatments at both time points to avoid extraction bias. For each RNA extraction batch and cDNA synthesis, we used blank controls to control for possible contamination from the kit and extraction process. In total, we generated 79 samples with successful RNA extraction followed by cDNA synthesis and six blank controls.

Samples were barcoded and sent to the Institute of Clinical Molecular Biology, Christian-Albrechts-University Kiel where they were sequenced for V1-V2 variable region of 16S rRNA gene.

### Immune Priming Treatments

#### Injection Priming Treatment

We generated overnight bacterial culture as previously described (Roth et al., 2009; Ferro et al., 2019) and adjusted the concentration to 1 × 10^9^ ml^–1^ bacteria followed by heat inactivation for 30 min at 90°C. To ensure a complete inactivation, a subsample of the heat-inactivated bacteria was cultivated on lysogeny broth (LB) agar plates at 30°C.

We primed 120 individuals of 15-day-old larvae with heat-killed *B. thuringiensis* [DSM no. 2046, German Collection of Microorganisms and Cell Cultures (DSMZ)] by injecting 18.4 nl of a 1 × 10^9^ ml^–1^ concentrated heat-killed bacterial dilution laterally between the 2nd and 3rd most posterior segment with the Nanoject II (Drummond Scientific Company, Broomall, PA, United States). The injected volume is equivalent to a dose of about 18,400 heat-killed bacteria per larva. We used 120 larvae as injection control (Injection Wounded) and injected them with 18.4 nl of sterile phosphate-buffered saline (PBS) (Calbiochem^®^) while 120 larvae were left untreated (naïve). We placed all injected and naïve beetles into 96-well plates individually containing flour with 5% yeast and kept them at normal rearing conditions until sampling.

#### Oral Priming Treatment

With some minor modifications, we prepared spore cultures and supernatants as previously described (Milutinović et al., 2014; Futo et al., 2016). We plated bacteria from frozen stock on LB agar plates and incubated overnight at 30°C. The next day we inoculated 5 different colony-forming units in 5 ml of *Bt* medium [w/V–0.75% Bacto Peptone (Sigma), 0.1% glucose, 0.34% KH_2_PO_4_, 0.435% K_2_HPO_4_] that was supplemented with 25 μl of sterile salt solution (0.2 M MgSO_4_, 2 mM MnSO_4_, 17 mM ZnSO_4_, and 26 mM FeSO_4_) and 6.25 μl of sterile 1 M CaCl_2_ × 2H_2_O, incubated at 30°C overnight. We transferred the overnight cultures to 1-L Erlenmeyer flasks consisting of 300 ml of *Bt* medium supplemented with 1.5 ml of salt solution, 375 μl 1 M CaCl_2_ × 2H_2_O. The cultures were incubated for 6 days at 180 rpm, 30°C. On the third day of incubation, we added 1.5 ml of salt solution and 375 μl 1 M CaCl_2_ × 2H_2_O to the cultures. On day six, we centrifuged the cultures at 4,500 rpm for 15 min at room temperature. The remaining supernatants from *Btt*, *Bttol*, and *Bt407*^–^ were centrifuged two times at 4,500 rpm for 15 min and then filter-sterilized with a 0.45-μm pore-size, followed by a 0.2-μm pore-size cellulose acetate filter (Whatman GmbH). We mixed every milliliter of spore-free supernatant with 0.15 g heat-treated flour (supplemented with 5% yeast). We pipetted 10 ml of the priming diet mixture into each well of a 96-well plate (Sarstedt, Germany), followed by sealing with breathable foil (Kisker Biotech) and drying overnight at 30°C. For the medium control treatment, the diet was prepared by mixing 0.15 g heat-treated, yeast-supplemented flour with each milliliter of non-conditioned *Bt* medium. To ensure that there were no live spores or bacteria, we cultivated filter-sterilized supernatants on LB agar plates and in LB medium at 30°C overnight. In total, we used 480 larvae for oral priming treatments. A number of 120 larvae per treatment were put individually on a previously prepared priming diet in 96-well plates. After 24 h on a diet, we sampled 60 larvae and transferred the remaining 60 onto control flour disks consisting of PBS mixed with 0.15 g/ml flour, in which they stayed for four days until they were sampled again.

#### Priming and Infection With *Btt* and *Bt tolworthi*

From the previous studies, we have the information of oral priming and infection abilities of *Btt* and *Bt407*^–^ in red flour beetles (Milutinović et al., 2014), but the same data are lacking for *Bt tolworthi.* Earlier research has shown that the *Bacillus* strain containing the same Cry toxin (Cry3Ba) as *Bt tolworthi* can induce mortality and priming response *via* oral route in beetle larvae (Futo et al., 2017). To investigate whether there would be the same outcome in *Bt tolworthi*, we performed a priming and infection experiment with *Bt tolworthi* and *Btt* as a positive control. Then, 15-day old larvae were exposed to *Btt*, *Bt tolworthi*, and medium control diet prepared as previously described. After 24 h on a diet, we transferred the larvae to the PBS flour disks and 4 days later exposed them to *Btt* or *Bt tolworthi* spores (5 × 10^9^ ml^–1^). Mortality was screened for 7 days and dead larvae were identified by immobility, the characteristic body shape, and a darkened color. We performed this experiment in a full factorial design, in two independent blocks each consisting of 48 larvae/treatment. All larvae were kept individualized in 96-well plates.

We prepared spore cultures for infection as previously described and as in Milutinović et al., 2013 with minor modifications. After 6 days of sporulation and the first centrifugation step, we washed the spores in 20 ml of PBS. After centrifugation, we resuspended the spores in 5–10 μl of PBS (depending on the size of the pellet) and counted them in the Thoma counting chamber. We adjusted the concentration to 5 × 10^9^ ml^–1^ spores and mixed them with 0.15 g of flour/ml of spore suspension. For negative control, we used PBS mixed with 0.15 g/ml flour. We pipetted 10 μl of either spore or control solution into 96-well plates, sealed them with the breathable foil, and dried them at 30°C for 24 h.

### Sample Processing

#### Sample Preservation

On the first day and fourth day post-priming, we removed 10 larvae from each treatment group from the flour disks and quickly transferred them into a 1.5-ml Eppendorf tube, which was immediately immersed in liquid nitrogen and stored at −80°C for further extraction. This resulted in six replicates per treatment group per time point.

#### RNA Extraction and cDNA Synthesis

We homogenized all frozen samples with a pestle in liquid nitrogen followed by adding 100 μl of phenol/chloroform/isoamyl alcohol (PCI) to each tube. Following the instructions suggested by the manufacturer, we isolated the microbial RNA using RNeasy PowerMicrobiome Kit (Qiagen, Hilden, Germany)/(MoBio). After checking the quality of purified RNA with gel electrophoresis and quantifying the RNA concentrations with the Qubit RNA HS Assay (Life Technologies, Thermo Fisher Scientific), we performed cDNA synthesis with SuperScript II reverse transcriptase (Life Technologies, Inc.) with random hexamers from the RevertAid cDNA synthesis kit (Fermentas). We stored all cDNA samples at –20°C until further processing. Upon quality check, five samples were excluded due to insufficient RNA concentrations for further processing. In total, 79 samples were obtained: 12 samples for the “Naïve” group (6 per time point), 12 samples for the “Injection_Wounded” treatment, 12 samples for “Injection_*Btt*” (6 per time point), 12 samples for “Oral_Medium” (6 per time point), 10 samples for “Oral_*Btt*” (5 per time point), 10 samples for “Oral_*Bt407*^–“^ (5 per time point), and 11 samples for “Oral_*Bttol*” (6 for first time point, 5 for second time point).

#### Library Preparation and 16S Sequencing

Library preparation and 16S sequencing were done at the Institute of Clinical Molecular Biology, Christian-Albrechts-University Kiel. For sequencing, variable regions V1 and V2 of the 16S rRNA gene within the DNA samples were amplified using the primer pair 27F-338R in a dual-barcoding approach as per the study of Caporaso et al. (2012). About 3.5 μl of cDNA was used for amplification, and PCR products were verified *via* the agarose gel electrophoresis. PCR products were normalized using the SequalPrep Normalization Plate Kit (Thermo Fischer Scientific, Waltham, MA, United States), pooled equimolarly, and sequenced on the Illumina MiSeq v3 2 × 300 bp (Illumina Inc., San Diego, CA, United States). Demultiplexing after sequencing was based on 0 mismatches in the barcode sequences.

### Analysis

#### Bioinformatics Processing

We processed all demultiplexed paired-end FASTQ files using the dada2 pipeline (v1.10.1) (Callahan et al., 2016) in R studio (v3.5.0). In summary, we filtered out low-quality sequencing reads and trimmed them to a consistent length. The truncLen option was set to 240 and 220 for forward and reverse reads, respectively. Additionally, the trimLeft option was set to five to trim the 5’ ends of the reads. Next, the filtered reads were dereplicated, and the learnErrors function was used to learn error rates for the amplicon dataset. Subsequently, the paired reads were merged to obtain a unique amplicon sequence variant (ASV) table, following the step in which chimeras were filtered out. Each ASV represents a unique 16S rRNA of various microbial strains. The taxonomic assignment of these ASVs was performed using the native Bayesian classifier of dada2 trained against the SILVA reference database (Pruesse et al., 2007) (release 138). Multiple sequence alignment was performed using the *DECIPHER* (v 2.10.2) (Wright, 2015) and phangorn (v2.5.5) (Schliep, 2011) packages followed by phylogenetic tree construction using FastTree (v 2.1.11) (Price et al., 2010). For further filtering, we used *phyloseq* (v1.26.1) (McMurdie and Holmes, 2013) and *decontam* (v1.2.1) (Davis et al., 2018) packages. All sequences that were significantly more prevalent in negative controls than in positive samples (threshold 0.5) were identified as contaminants (310) and were removed from the dataset. Additionally, we also removed cyanobacteria as they most likely came from chloroplast reads from the flour.

#### Statistical Analysis

All statistical analyses and plots were produced using the web tool Microbiome Analyst (Dhariwal et al., 2017; Chong et al., 2020) and R studio (v3.5.0). A gene tree, ASV taxonomy, ASV counts, and sample table (Supplementary Table 1) were generated using phyloseq package and further analyzed using Microbiome Analyst. Using this web tool, we calculated and plotted relative abundances, calculated observed species richness and Shannon biodiversity index (Hill et al., 2003), and performed principal component analysis (PCA) (Gower, 2015) and linear discriminant analysis (LDA) effect size (LEfSe) (Segata et al., 2011). Before analysis, we filtered out ASVs that had mean abundance values less than minimum ASV read counts and with less than four reads.

Further analysis and plots of the observed species richness and Shannon biodiversity index were performed in R studio. To test for a normal distribution of the indices, a Shapiro–Wilk test was performed (Shapiro and Wilk, 1965). Since the observed species richness and Shannon index were normally distributed, and assumptions were met (homogenous variation between treatments), one-way ANOVA was performed. The means were compared using Tukey’s honest significant differences (HSDs).

Before performing PCA, data were normalized using total sum scaling (TTS) normalization. We visualized relative composition per treatment using PCA based on Bray–Curtis dissimilarity and employed PERMANOVA (a permutational ANOVA/MANOVA) and PERMDISP (permuted dispersion, which tests for homogeneity of dispersions) to statistically evaluate the treatment effect.

To determine the taxa driving the differences between the treatments, we performed biomarker discovery with LEfSe. Before LEfSe analysis, relative log transformation (RLE) of the data was done. We identified significant taxa based on the FDR adjusted to cutoff < 0.05 and LDA score < 2.0. We used BLAST^1^ to identify the sequences of the taxa that were significantly differentially abundant.

For the analysis of survival, a Cox proportional hazards model was applied with one random effect (Therneau et al., 2003) using coxph function from the “survival” package (Therneau, 2021). The treatment was defined as the fixed factor, while a putative plate effect was defined as a random factor and added as a frailty term. We checked the necessary assumptions for survival analysis by checking Schoenfeld’s residuals to test whether hazards are proportional and whether we have influential cases in the data. The assumptions were met, and after fitting the model, the variance between treatments was assessed using a one-way ANOVA. The means were compared using Tukey’s *post hoc* analysis with Benjamini–Hochberg correction.

## Results

### Microbiome Analysis and Composition

In the red flour beetle *T. castaneum*, oral immune priming through the ingestion of sterile bacterial spore culture supernatants has been shown to depend upon the presence of the larval microbiome (Futo et al., 2016). Here, we investigated whether different priming treatments lead to a difference in larval microbiome diversity. Microbiome composition was assessed by deep sequencing of the V1-V2 region of the bacterial 16S rRNA gene, at two time points, 24 h and 4 days after the priming treatments.

**FIGURE 2:**
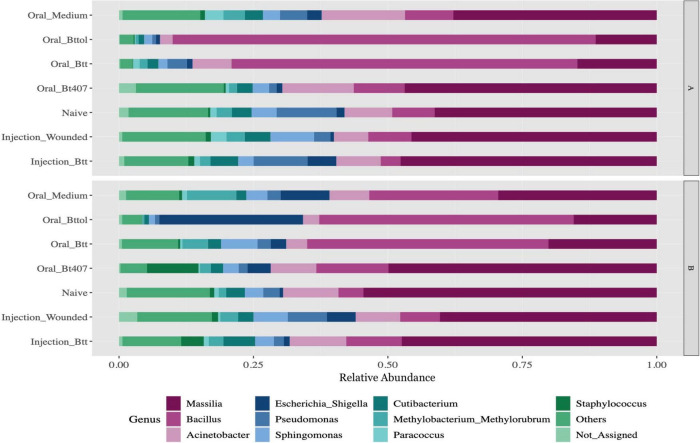
Relative microbiome composition of red flour beetle larvae based on bacterial 16S rRNA gene profiling after seven different priming treatments in two time points (A: 24 h after priming, B: 4 days after priming). The y-axis indicates relative abundances (sums to 1 for each treatment) of all the ASVs detected. Each treatment corresponds to 12 replicates of 10 pooled larvae each. Only top 10 genus have been represented in the figure with all other genus being aggregated as Others. Not_Assigned represents all ASVs for which genus could not be assigned. For the absolute microbiome abundance, see Supplementary Figure 4.

After quality filtering and processing, we retained in total 4,203,008 16S rRNA reads with an average of 53,202 reads per sample across 79 samples. After filtering out low count ASVs for both time points together, a total of 2,047 low abundance ASVs were removed and 441 ASVs remained. Overall, the 10 most abundant ASVs of the larvae microbiome matched to the genus *Massilia* (35%), *Bacillus* (26%), *Acinetobacter* (8%), *Escherichia-Shigella* (5%), *Pseudomonas* (3%), *Sphingomonas* (4%), *Cutibacterium* (3%), *Methylobacterium_Methylorubrum* (3%), *Paracoccus* (1%), and *Staphylococcus* (2%) (Figure 2 and Supplementary Figure 1). The relative abundance plot suggests that the abundance of the *Bacillus* genus is higher in oral priming treatments with *Btt* and *Bt tolworthi* while the microbiome of the other treatments seems relatively stable. Both time points overall showed a similar microbiome composition (Supplementary Figure 2).

### Oral Priming Treatments Modify Microbiome Diversity

**FIGURE 3:**
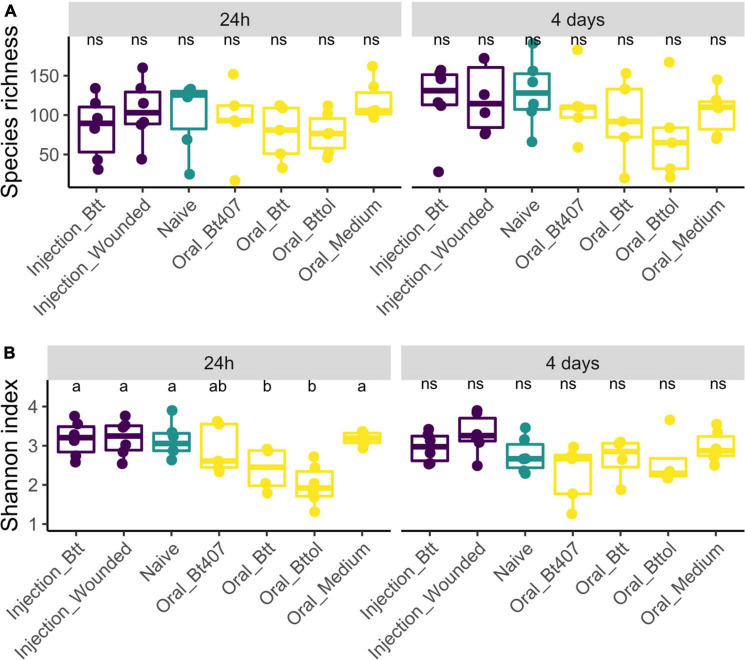
Alpha biodiversity measures in relation to time points and treatment regimes. Each dot represents one replicate of 10 pooled larvae. The letters indicate the differences between the treatments after *post hoc* and *p*-value adjustment. **(A)** Observed species richness measure of experimental treatments 24 h and 4 days after priming shows no difference between the regimes (24 h: ANOVA, *F* = 0.95, Df = 6, *p* = 0.47; 4 days: ANOVA, *F* = 0.90, Df = 6, *p* = 0.50). **(B)** Shannon index measures of experimental treatments 24 h after priming shows a higher diversity in oral *Btt* and oral *Bt tolworthi* treatments (ANOVA Df = 6, *F* = 5.92, *p* = 0.003) and 4 days after priming showing no difference between the treatments (ANOVA, *F* = 2.30, Df = 6, *p* = 0.060).

We analyzed the microbiome diversity measures for the two time points separately (24 h: ANOVA, *F* = 0.95, Df = 6, *p* = 0.47; 4 days: ANOVA, *F* = 0.90, Df = 6, *p* = 0.50), which results in 352 and 461 ASV features for the first and second time points, respectively. We used observed species richness and Shannon biodiversity index to evaluate whether the different priming treatments result in differences in the microbiome diversity. Observed species richness was consistent in different treatment regimes and time points. However, larvae treated with *Btt* and *Bt tolworthi* supernatants showed significantly lower diversity in the Shannon index at the first time point (ANOVA, *F* = 5.92, Df = 6, *p* = 0.003) compared to all other treatments while no difference was detected at the second time point in any of the treatments (ANOVA, *F* = 2.30, Df = 6, *p* = 0.06) (Figure 3).

**FIGURE 4:**
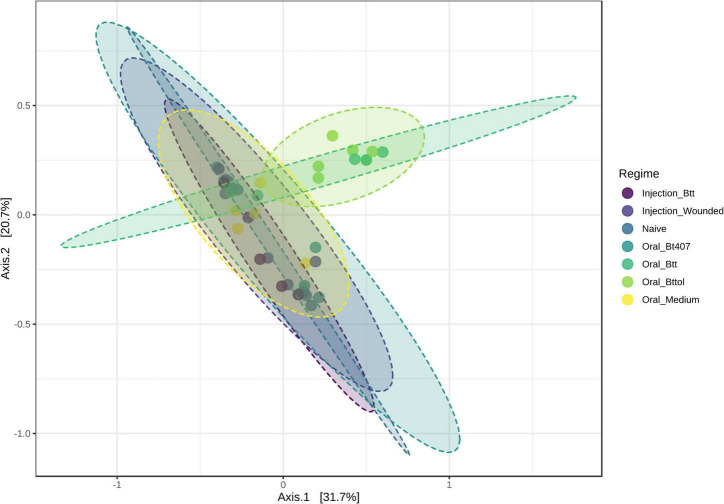
Principal—coordinate (PCoA) plot of the first time point (24 h after priming) based on Bray—Curtis dissimilarity distances. Priming with *Btt* and *Bt tolworthi* have an impact on the microbiome composition. Ellipses are drawn around samples belonging to the same priming regime and there are six replicates of each of the regimes. Permanova: *F* = 2.179, *R*^2^ = 0.284, *p* < 0.001. Perdmadisp: *F* = 2.170, *p* = 0.071.

To investigate the impact of priming treatments on the microbiome community structure, we conducted principal coordinate analysis (PCoA) using Bray–Curtis dissimilarity distances (Supplementary Table 2) for both time points. Only for the first time point (24 h after priming), the diversity measures showed differences between priming treatments. Our analysis revealed that samples belonging to larvae orally primed with *Btt* and *Bt tolworthi* cluster closely together indicating that those two priming regimes impact the larval microbiome (Figure 4; PERMANOVA, *F* = 2.1791 *R*^2^ = 0.28377, *p* < 0.001). PERMDISP analysis showed not to be significant which means that there was no difference in dispersion between groups, and the difference detected is coming from *Btt* and *Bttol* priming treatments (PERMDISP: *F* = 2.1699, *p* = 0.071298). Additional analysis of the second time point (4 days after priming) showed that there is no impact of different priming treatments on the microbiome composition of the larvae (Supplementary Figure 3).

### Taxa Belonging to the Genus of *Bacillus* Are Responsible for the Microbial Change Following Oral Priming Treatments

**FIGURE 5:**
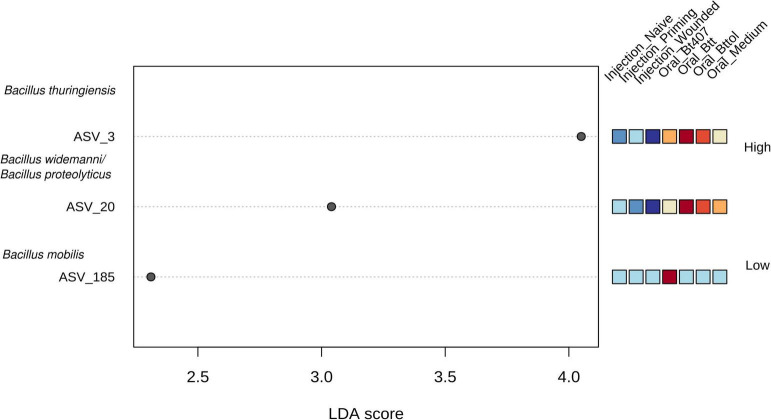
Linear discriminant analysis effect size plot. Significant features are ranked in decreasing order by their LDA scores (x-axis). The heatmap to the right of the plot indicates whether the taxa are higher (red) or lower (blue) in each treatment. FDR < 0.05, LDA score >2. Using BLAST all ASVs were identified as *Bacillus*.

Next, we identified the features (ASVs) that are responsible for observed diversity differences in the first time point (24 h after priming) with a LEfSe analysis (Supplementary Table 2). A number of three taxa—ASV 3, ASV 20, and ASV 185—were identified as significant (Figure 5; FDR < 0.05, LDA > 2). ASV 3 and ASV 20 were found to be the most abundant in “Oral_*Btt*” and “Oral_*Bttol*” priming treatments, whereas ASV 185 seemed to be present only in “Oral_*Bt407*” treatment. Since we did not get a taxa match for the significant ASVs when searching against SILVA database, we searched their nucleotide sequences in BLAST and identified that ASVs in question belong to different species of *Bacillus* (*Bacillus thuringiensis* as ASV 3, *Bacillus widemanni/Bacillus proteolyticus* as ASV 20, and *Bacillus mobilis* as ASV 185). However, the discrimination between *Bacillus* species based on 16S rRNA gene is unreliable (Chen and Tsen, 2002; Soufiane and Côté, 2009). Therefore, with these results, we suspect that bacterial cues in the priming diets with *Btt* and *Bt tolworthi* strains increased the abundance of only certain members of the *Bacillus* genus and thereby have a strong effect on the microbiome community of the beetle larvae.

### *Bt tolworthi* Does Not Induce a Priming Response in the Flour Beetle Larvae

**FIGURE 6:**
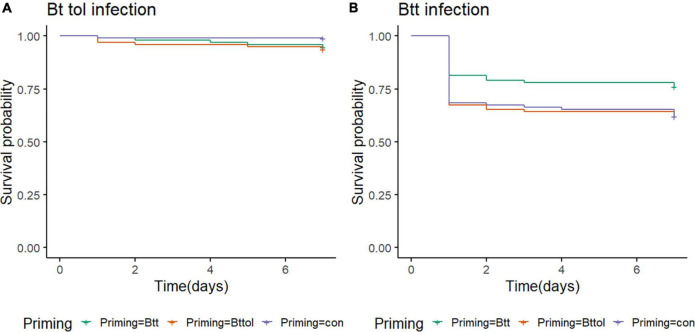
*T. castaneum* survival upon different priming treatments. **(A)** There is no increase in survival of 19-day-old beetle larvae orally infected with *Bt tolworthi* spores (5 × 10^9^ mL^–1^) when previously primed with *Btt* or *Bt tolworthi* supernatants [Cox proportional hazards (coxph): *X*^2^ = 4.74, Df = 1.96, *p* = 0.09]. **(B)** Survival of 19-day-old beetle larvae orally infected with *Btt* spores (5 × 10^9^ mL^–1^) is increased when previously primed with *Btt* supernatant [Cox proportional hazards (coxph): *X*^2^ = 5.9, Df = 2, *p* = 0.05]. 5 days prior to the spore infections, larvae were exposed to priming diet mixture with flour and either sterile supernatants of *Btt*, *Bt tolworthi* or control *Bt* medium.

We observed similar changes in microbiome after oral priming treatment with *Btt* and *Bt tolworthi.* This raises the question of whether (1) *Bt tolworthi* is able to kill *T. castaneum* larvae upon oral exposure, and (2) whether it also leads to the protection through a priming response. The previous experiments have shown that mortality in *T. castaneum* larvae results from ingestion of *Btt* spores (harboring the Cry3Aa toxin), but also from another *Bacillus* strain that has a Cry3Ba toxin and bacterial cues which induced a specific priming response orally (Futo et al., 2017). *Bt tolworthi* also carries a Cry3Ba toxin, but it has not yet been investigated whether this strain is able to induce a priming response in red flour beetles. Therefore, we conducted oral priming and challenge experiments with *Btt* and *Bt tolworthi* in a full factorial design. After 7 days of tracking survival, we observed that *Bt tolworthi* did not induce any mortality or priming response in red flour beetle larvae (Cox proportional hazards (coxph): *X*^2^ = 4.74, Df = 1.96, *p* = 0.09) (Figure 6A). Moreover, exposure to bacterial cues from *Bt tolworthi* did not contribute to improved survival against *Btt* infection (i.e., no unspecific priming effect) (Figure 6B). By contrast, in the same experiment, we could confirm the previously reported (Milutinović et al., 2014; Futo et al., 2017) priming response in larvae treated with *Btt* [Cox proportional hazards (coxph): *X*^2^ = 5.9, Df = 2, *p* = 0.05] (Figure 6B).

## Discussion

While there is increasing evidence for the role of the microbiome in the formation of host immune responses, potential interactions of the microbiome with immune priming mechanism in invertebrates are currently less clear. To our knowledge, this is the first study to show that different immune priming treatments affect the insect hosts’ microbiome composition. Using a well-established model for immune priming, the red flour beetle *T. castaneum* primed with the entomopathogenic *B. thuringiensis*, we found that oral immune priming treatments of beetle larvae significantly changed their microbiome diversity and composition, as determined *via* 16S rRNA sequencing. By contrast, septic priming treatment *via* injection did not change the larval microbiome. Interestingly, the observed changes in microbial communities were largely driven by an increased abundance of *Bacillus* bacteria, that is, species belonging to the same genus as the entomopathogen used for the priming treatments.

The most abundant bacterial taxa in whole body larvae across all treatments belong to the phyla Proteobacteria, Firmicutes, and Actinobacteria, and the genera *Bacillus, Acinetobacter, Pseudomonas*, and *Escherichia*. These microbiome compositions are consistent with the previous studies in insects in general (e.g., Yun et al., 2014), as well as the studies in *T. castaneum* (Kumari et al., 2011; Agarwal and Agashe, 2020). However, while *Enterobacteriaceae* were the most common and dominant bacteria in adults, larvae, and pupae of flour beetles in Agarwal and Agashe (2020), the genus *Massilia*, which has so far not been described for *T. castaneum*, was most prevalent in most of our treatments. *Massilia* is considered to be a part of the soil microbiome (Ofek et al., 2012) and has been reported in black ants (Osimani et al., 2018) and longhorn beetles (Rizzi et al., 2013). Some members of this genus show endochitinase capabilities (Adrangi et al., 2010) which could be important for cuticle organization after molting or degradation of an old cuticle in *T. castaneum* (Noh et al., 2018).

The oral priming treatment, that is, feeding of sterile spore culture supernatants of *B. thuringiensis* resulted in differences in microbial diversity and composition 24 h after treatment, which were not detectable anymore at a later time point 4 days after treatment. As these differences were driven by an increased abundance of *Bacillus* species, we had to exclude any direct contamination from the priming diet. Priming diets were filter-sterilized to remove all bacterial cells, and additional plating and incubation in the LB medium showed no bacterial growth. Furthermore, we consider it unlikely, but cannot fully exclude, that RNA traces present in the supernatant may have directly contributed to the observed expansion of *Bacillus* species in larvae exposed to these diets, because we could not detect any 16S rRNA above background level (i.e., medium control; Supplementary Figures 5, 6), which corresponds to the RNA concentration lower than the 1:10,000,000 dilution of the *Btt* RNA positive control with 450 ng/μl of RNA.

It is thus most likely that *Bacillus* species that were previously present in the larvae expand in response to the priming treatment. Indeed, *Bacillus* has been previously reported in *T. castaneum* (Kumari et al., 2011; Agarwal and Agashe, 2020) and also in the closely related *Tenebrio molitor* (Osimani and Aquilanti, 2021). Furthermore, *Bacillus* was present in all our treatments, not only the orally primed ones. What could cause increased growth of resident *Bacillus* after the oral priming treatment? Cues or metabolites in the spore culture supernatant could trigger growth and enhance the competitive success of *Bacillus*. Filter-sterilized supernatants from *Bacillus* species were shown to consist of proteins and peptides, metabolites, quorum-sensing-related proteins, microbial wall cell components, and flagellins (Gohar et al., 2005). Secreted peptides of *Bacillus* can serve as quorum-sensing molecules (Slamti et al., 2014). Along with quorum-sensing-related proteins, other metabolites and even monomers of the Cry proteins could potentially contribute to the growth of *Bacillus* in orally primed larvae.

Could the increased abundance of *Bacillus* also cause the priming effect, that is, higher survival upon challenge? Indeed, higher *Bacillus* abundance could cause an immune activation that prepares the larvae for defense against the following infection. The previous transcriptome studies of orally primed *T. castaneum* indeed identified the upregulation of several genes involved in defense against oral pathogens, such as hexamerin, pathogenesis-related protein 5, lysozyme, and hdd1 defense protein (Greenwood et al., 2017), as well as long non-coding RNAs (lncRNAs), which potentially control immune responses (Ali and Abd El Halim, 2020; Shirahama et al., 2020).

However, we observed that similar microbiome changes in larvae treated with *Btt* and *Bt tolworthi* may argue against a direct causal role of the microbiome for oral immune priming, because contrary to *Btt*, *Bt tolworthi* neither orally primes nor kills *T. castaneum*. *Bt tolworthi* carries a different Cry toxin than *Btt*, namely, Cry3Ba. Even though this toxin has a high affinity to receptors of the midgut epithelial cells of *T. castaneum* (Contreras et al., 2013) and another *Bt* strain carrying Cry3Ba caused mortality and priming (Futo et al., 2017), *Bt tolworthi* did not induce significant mortality or priming in the present and previous studies (Milutinović et al., 2013; Zanchi et al., 2020). LEfSe analysis showed that one taxon belonging to *Bacillus* is more abundant also in the *Bt407*^–^ priming treatment. This could be explained by previously mentioned quorum sensing where secreted peptides in the supernatant could facilitate the growth of resident *Bacillus*. While *Bt407*^–^ is lacking any, both *Btt* and *Bt tolworthi* express Cry proteins, and it is possible that monomers of the protein remain in the filter-sterilized supernatant and could even further promote the communication and growth of the existing *Bacillus* strain.

Even though taxa that were driving the difference among the oral treatments were identified using BLAST on a species level, we are careful in concluding that those three different ASVs belong to different species and only consider that ASVs in question belong to the genus *Bacillus.* Due to the high conservation of the 16S rRNA gene, differentiation between the *Bacillus cereus* and *Bacillus thuringiensis* strains is unreliable and many authors suggest using additional methods to discriminate between the strains (Chen and Tsen, 2002; Soufiane and Côté, 2009). The potential presence of two or more 16S sequences within a single *Bacillus* genome (e.g., Prüss et al., 1999) may suggest that ASV 3 and ASV20, which appear in roughly similar proportions across our different samples, might even originate from one and the same *Bacillus* strains. This question could in the future be tackled with long-read sequencing technology (Johnson et al., 2019).

Priming treatment *via* injection with heat-killed *Btt* does not influence the *T. castaneum* microbiome compared to injection control and naïve larvae. Such a difference to oral priming could arise if at least partially separate mechanisms underpin immune priming *via* the two different routes. This is not unlikely, as host defenses depend on the infection routes of pathogens, and different immune parameters are activated in the hemolymph and gut (e.g., Martins et al., 2013). For *T. castaneum*, transcriptomic data showed that the gene activation greatly differs between the routes of *B. thuringiensis* infection (Behrens et al., 2014). The septic route brings a pathogen into direct contact with immune cells and effectors in the hemocoel, whereas special adaptations are needed for pathogens to break the gut barrier. Accordingly, different priming mechanisms could enable the protection of the host when it comes into contact with a priming agent through these different routes. Candidate mechanisms for priming vary across different insect host species and priming routes (Milutinović and Kurtz, 2016), although we currently lack detailed knowledge of route-specific priming mechanisms within any one species, including *T. castaneum* (Milutinović et al., 2016). What could be the role of the microbiome for immune priming, in the light of the present and previous study showing that priming disappears in the absence of the microbiome (Futo et al., 2016)? We suggest that oral immune priming in *T. castaneum* may depend on the interplay of host immune factors with members of the resident microbiome and the pathogen itself. Any direct role of the gut microbiota for an unspecific immune priming effect *via* hemolymph factors as observed in *Anopheles gambiae* (Rodrigues et al., 2010; Gomes et al., 2021) is unlikely in our system because it could not explain the observed bacterial-strain specificity of the oral priming response (Futo et al., 2017). A study in *Drosophila* revealed that antiviral resistance is achieved by a two-signal system: microbiota-dependent priming and virus-dependent sensing (Sansone et al., 2015). Something similar might happen in our system where the oral priming response could depend on resident microbiome changes and sensing that is dependent on bacterial cues.

Even though the details of the mechanisms behind priming still remain unclear, this study yields further insight into the interplay of different immune treatments with the microbiome of invertebrates. To better understand the specific role of members of the microbiome in priming responses, it would be important to experimentally manipulate the microbial communities. This would help to further deepen our understanding of such forms of memory in invertebrate immunity.

## Data Availability Statement

The sequencing data generated for this study have been submitted to the NCBI BioProject database and can be accessed under the accession number: PRJNA765158. The scripts used to process the sequencing data can be accessed via GitHub repository using the following link: https://github.com/shreygandhi1990/immune_priming_microbial_composition.

## Author Contributions

AK and JK conceived and designed the experiments. AK and LL performed the experiments. CB sequenced the samples retrieved from the experiment. AK and SG analyzed the data. AK wrote the original manuscript draft. AK, SG, LL, JK, and CB wrote and revised the manuscript and approved the final version for publishing.

## Conflict of Interest

The authors declare that the research was conducted in the absence of any commercial or financial relationships that could be construed as a potential conflict of interest.

## Publisher’s Note

All claims expressed in this article are solely those of the authors and do not necessarily represent those of their affiliated organizations, or those of the publisher, the editors and the reviewers. Any product that may be evaluated in this article, or claim that may be made by its manufacturer, is not guaranteed or endorsed by the publisher.

## Footnotes

1
https://blast.ncbi.nlm.nih.gov/Blast.cgi
