# Supplementary material for: Oral Immune Priming Treatment Alters Microbiome Composition in the Red Flour Beetle Tribolium castaneum
Source: Front Microbiol. 2022 Apr 13;13:793143. doi: 10.3389/fmicb.2022.793143 (PMC9043903; doi:10.3389/fmicb.2022.793143)
Supplement: Supplementary file 1 [file Data_Sheet_1.pdf]

Supplementary Material

1 Supplementary Data

ASV taxa, ASV count and Sample table used for analysis in Microbiome analyst

2 Supplementary Figures and Tables

2.1 Supplementary Figures

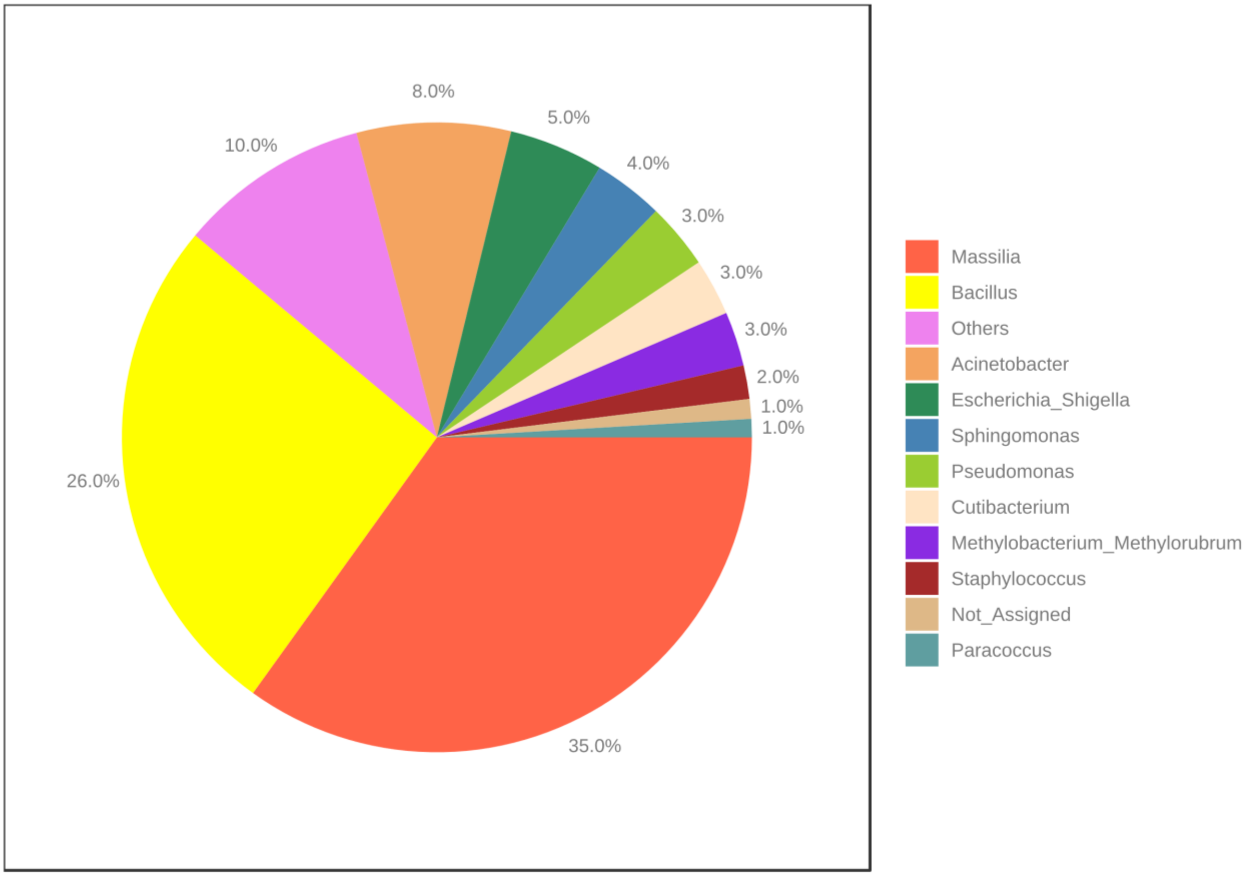

Figure S1: Pie chart diagram showing the percentage of taxa on the genus level found across samples in all treatments combined.

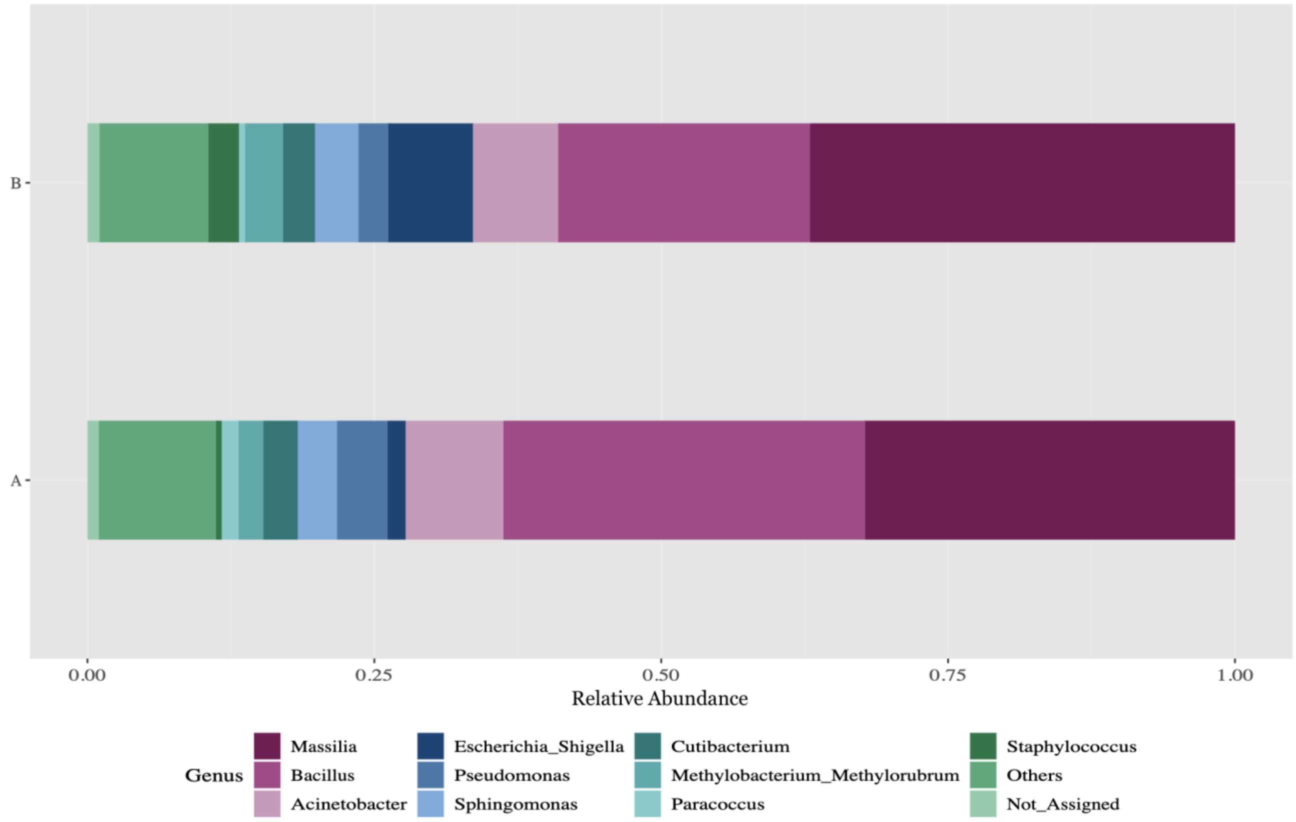

Figure S2: Relative microbiome abundance of the red flour beetle larvae based on bacterial 16S rRNA gene profiling at two time points. Time point A stand for 24h after priming treatment, time point B stands for 4 days after priming treatment. Both time points combine all the treatments together. The y-axis indicates relative abundances (sums to 1 for each treatment) of all the ASVs detected. Only top 10 genera have been represented in the figure with all other genera being aggregated as others. Not_Assigned represents all ASVs for which genus could not be assigned.

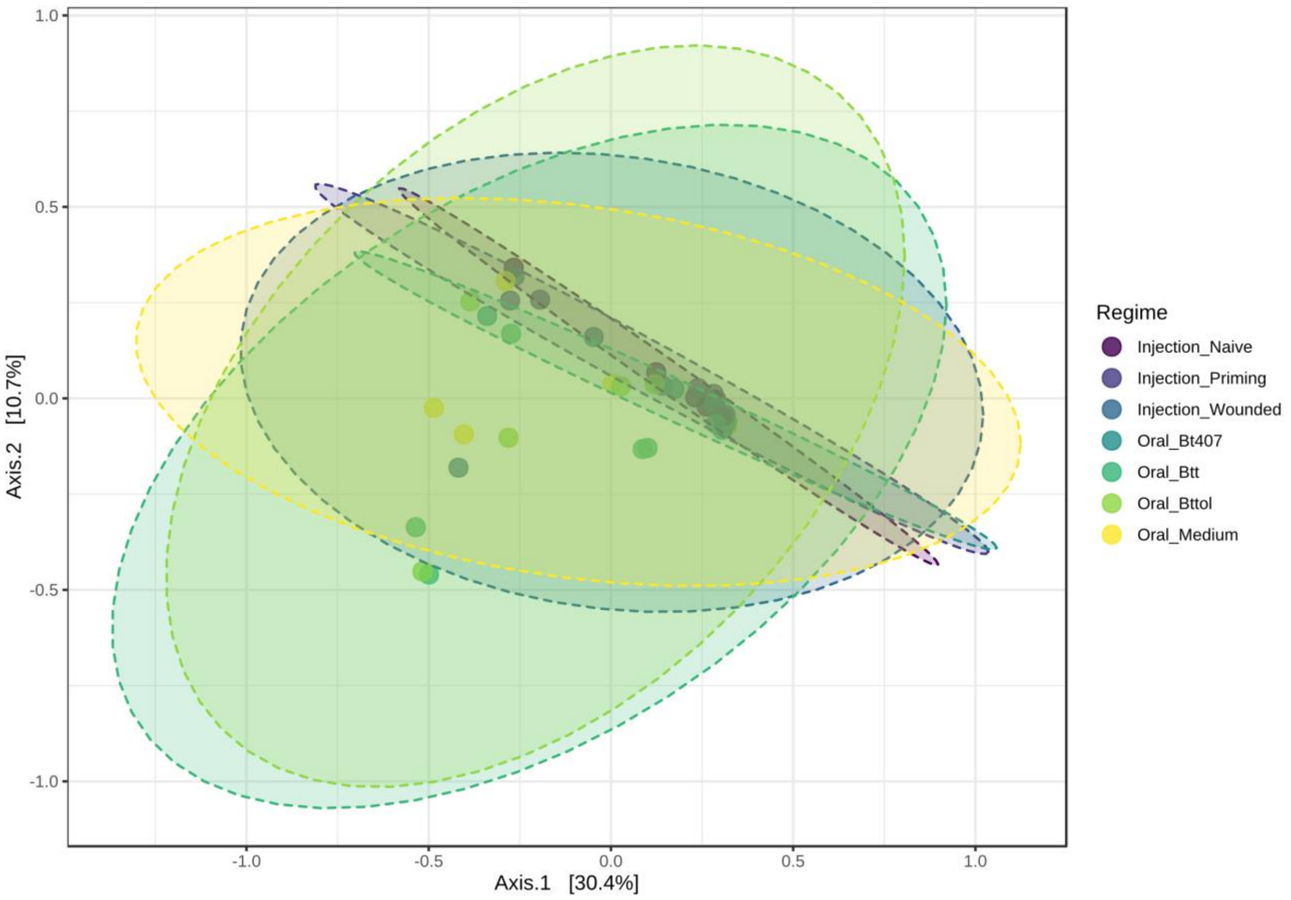

Figure S3: Principal – coordinate (PCoA) plot of the second time point (4 d) based on Bray – Curtis dissimilarity distances. The analysis was done with prior total sum scaling (TTS) normalization. Ellipses are drawn around samples belonging to the same priming regime. There are six replicates of each of the regimes.

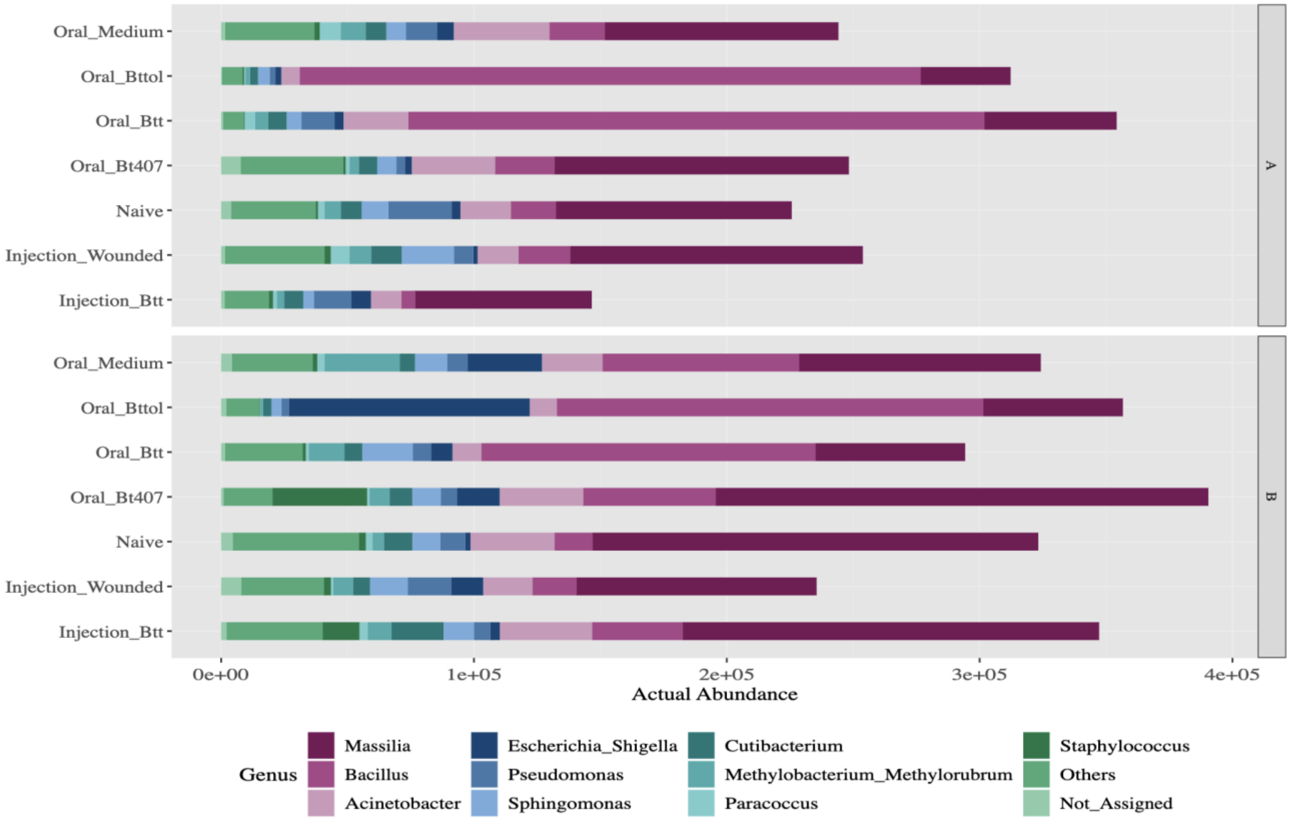

Figure S4: Absolute microbiome abundance of red flour beetle larvae based on bacterial 16S rRNA gene profiling after seven different priming treatments in two time points (A: 24h after priming, B: 4 days after priming). The y-axis indicates absolute abundances of all the ASVs detected. Each treatment corresponds to 12 replicates of 10 pooled larvae each. Only top 10 genus have been represented in the figure with all other genus being aggregated as Others. Not_Assigned represents all ASVs for which genus could not be assigned.

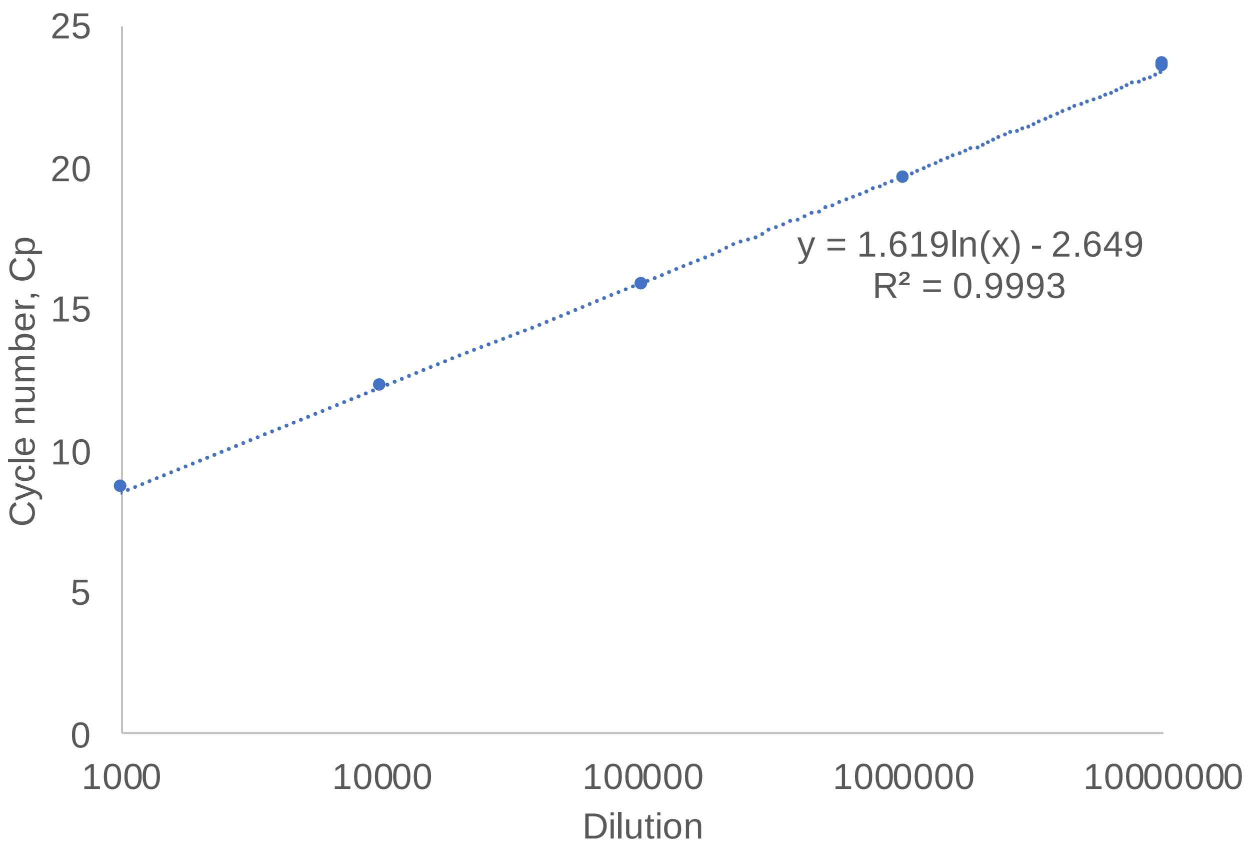

Figure S5. Calibration curve for quantification of 16S rRNA. The standard curve was formed using a dilution series from 1:1000 to 1:10000000 dilution (in steps of 10-fold dilution) of RNA extracted from the *Bacillus thuringiensis tenebrionis* (*Btt*) bacterial culture, which originally has a RNA concentration of 450 ng/µl. Primers used to amplify the hypervariable region V5-V6 of the bacterial 16S rRNA cDNA were fwd: 799F-mod2 (5′ AACMGGATTAGATACCCKGGT 3′) and rev: 1114R (5′ GGGTTGCGCTCGTTGC 3′) yielding a PCR product of approximately 315 bp.

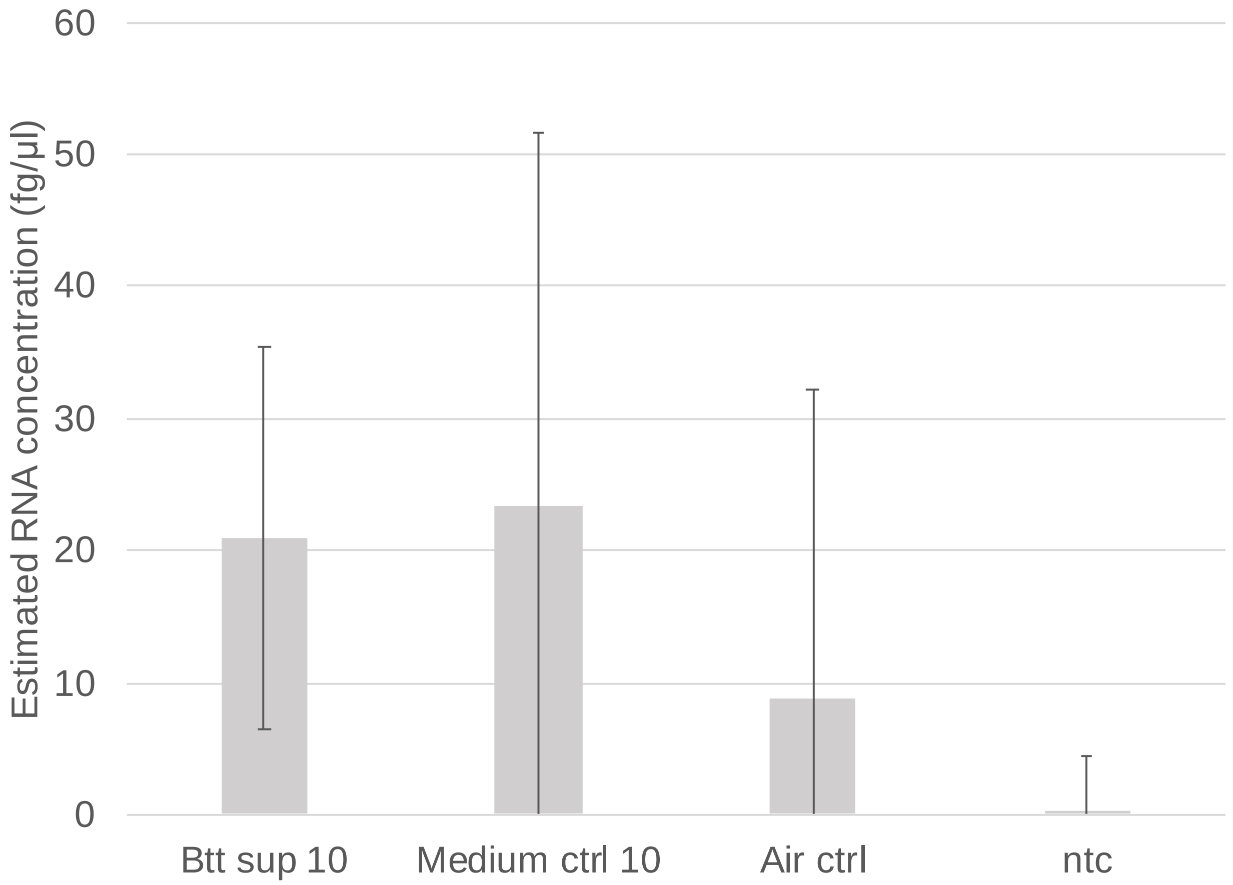

Figure S6. Mean estimated concentrations of total 16S rRNA across *Bacillus thuringiensis tenebrionis* (*Btt*) supernatant samples and controls. RNA amounts were estimated from the 10-fold serial dilutions of RNA extracted from *Btt* bacterial culture (Fig. S5). Each bar represents 3 replicates (each consists of technical duplicates) of the following samples: "*Btt* sup 10" = RNA sample extracted from 10µl of *Btt* supernatant; "Medium ctrl 10" = RNA sample extracted from 10µl of medium control; "Air ctrl" = control for RNA extraction; "ntc" = no template control for qPCR. The volume of supernatant or medium control used here corresponds to the volume (10 µl) of supernatant- or medium-flour mixture fed to each individual larva in all oral treatments. The graphs show group mean plus standard deviation of the mean.
